# Notes on a Dietetic Case

**Published:** 1888-09

**Authors:** 


### NOTES ON A DIETETIC CASE.

As an appendix to "A Dietetic Case," given in your July issue, page 345: Years ago I treated a young lady student of a very hysterical character, for a kind of gastric fever which would not yield to the usual treatment, and I might just as well acknowledge that this young student was as well versed in the intricacies of our materia medica as many an old head. That she was a thorough high-potency woman may be considered understood. One day at my morning visit she said to me: "Professor, what I have craved for the last three days, I know I ought not to have and still I want it badly and nothing tastes good." She wanted mushrooms. I immediately telegraphed the whole state to Constantine Hering, and after a few minutes the wire flashed back: "Let her have as many as she wishes and nothing else." My fair patient luxuriated on a can of mushrooms a day (it was in the midst of winter) and from that moment convalescence set in without another drop of medicine.

2. With the aid of two other physicians I treated once a very severe case of typhoid fever, which had run already four weeks without abating. On a Saturday afternoon our consultation resulted in the consolation for the parents that the result is more than doubtful. Sunday morning came and the old people went to church to pray for their daughter's restoration and left our poor patient to the care of an older sister. I had seen her about eight A. M. (she lived about six miles from my office). Cornbeef and cabbage was prepared for dinner, and the dear girl smelling the perfume arising therefrom, begged her sister to give her a taste. A refusal did not abate her desire, and the nurse finally yielded, thinking she might as well have her last wish on earth granted. It tasted so good to her, so refreshing. When the parents returned and heard this, messengers were immediately dispatched for the doctors to hurry up, and by good luck I was the first to arrive and found my fair patient enjoying the first sweet natural sleep and quiet breathing. At my advice we sat down to dinner and regaled ourselves on that cornbeef and cabbage, when Doctor No. 2 drove up and asked immediately whether I had given her already an emetic. "Hold your horses," I replied, and would not allow him to go to the sick-room till his impetuosity had calmed down. Doctor No. 3 came, and we smoked our pipes in unison and in peace and waited till six P. M., when she awoke refreshed as out of a dream. In short, the crisis was happily passed, and except a few doses of Cinchona for her weakness she took no more medicine and recovered nicely.

A queer diet this was, cabbage for typhoid fever; but my friend Kellogg, of New York, insists upon it that when a patient demands for days an unreasonable article of diet, let him have it, even if it is sour krout, and in many cases it is really a hint of nature which should command our best attention. S. L.

